# The pursuit of equity in COVID-19 policy and policymaking: A qualitative systematic review

**DOI:** 10.12688/openreseurope.20563.1

**Published:** 2025-06-18

**Authors:** Paul Cairney, Stefano Intropido

**Affiliations:** 1 Division of History, Heritage, and Politics, University of Stirling, Stirling, Scotland, FK94LA, UK

**Keywords:** COVID-19, policy, policymaking, inequalities, equity

## Abstract

**Background:**

The COVID-19 pandemic produced a devastating and unequal effect on global population health and wellbeing. Although research demonstrated multiple COVID-19 inequalities, the pursuit of equity (to address unfair inequalities) remained politically contested and overshadowed by higher priority crisis responses. It is essential to learn from these experiences to inform future crisis responses and anticipate the lack of proportionate and sustained attention to inequalities. We seek to understand how COVID-19 equity research defines this policy problem, offers solutions, and considers their feasibility in complex and political policy processes.

**Methods:**

We conducted a qualitative systematic review (2024) to identify peer reviewed journal articles on COVID-19, policymaking, and equity in three databases (Web of Science, Scopus, Proquest). We sought articles providing a non-trivial reference to policymaking concepts, including 55 texts that meet the inclusion criteria, and adding 30 texts by snowballing. We used an immersive and inductive approach to identify key themes and show how the use of policy concepts and theories informs an overall narrative of COVID-19 equity research.

**Results:**

This research documents the unequal impact of the COVID-19 pandemic and policy, then identifies potential policy solutions and some hopes that governments will support them. However, it highlights a major gap between this aspiration for change versus political reality, and identifies barriers to the production and use of lessons for future crises.

**Conclusion:**

Most governments appear to have learned few lessons about inequalities from their COVID-19 experiences. The literature suggests that most governments will contribute to very similar unfair inequalities in their responses to the next crisis.

## Plain language summary

COVID-19 produced a devastating effect on global population health and wellbeing. Many governments seemed underprepared, taking took too long to address the profoundly unequal impact of COVID-19. It is essential to use research on these experiences to learn to respond more effectively to the next crisis.

Policy learning seems like a valence issue: who would *not* want to learn to improve policy and policymaking? However, meaningful engagement between researchers and policymakers, foster such learning, is not guaranteed. A wealth of equity research highlights the size of the policy problem, and proposes some solutions, but without incorporating insights from policy process research. Research on solutions highlights the success of some governments, but without explaining why they are successful, or how to transfer success. Few studies acknowledge the politics of policy learning. Research on policymaking shows that inequalities are politically contested problems: there is high disagreement on how to define equity and address inequalities, as well as a major gap between required and actual levels of cooperation inside and outside of government. Further, research on science advice to policymakers, and evidence-informed policymaking, highlights routine gaps between the supply and use of research to inform policy.

In that context, we seek insights on how to understand three key components: COVID-19 inequalities, the pursuit of equity, and the potential to generate usable lessons for policymaking. We build on previous reviews that highlight contestation to define and pursue equity and a tendency for equity policies to be overshadowed by higher priority economic problems and security-focused emergency responses. It is essential to learn how to inform future crisis responses without assuming that such an important problem will receive proportionate attention or support. Rather, we find a strong possibility that most governments will not learn how to respond more effectively and equitably next time.

## Introduction

We present a qualitative systematic review of COVID-19 equity policy research. We focus on how researchers and policymakers define unfair inequalities and seek to improve equity, and what governments can learn about reducing inequalities when planning for the next crisis. This is the seventh *Open Research Review* conducted by our team, and we use insights from the review series to guide our questions, methods, analysis, and expectations. This consolidated approach allows us to frame this review with reference to the simple contrast between the following two perspectives.

The first perspective identifies a series of reasonable aspirations for policy and policymaking. The COVID-19 pandemic produced a devastating and unequal effect on population health and wellbeing, and it took many governments too long to recognise and respond to inequalities. COVID-19 inequalities exhibited key elements of ‘wicked’ problems, characterised by high uncertainty and ambiguity about their nature and cause, contestation over the feasibility of responses, and their tendency to transcend traditional policy sector boundaries, prompting the urgent need for substantive collaboration across and outside of government to produce proportionate and effective solutions ( Auld *et al.*, 2021; Cairney *et al.*, 2022a; Rittel & Webber, 1973). However, too few governments were up to the task. Therefore, it is essential to use research on positive and negative experiences to inform learning to improve the next response. Learning should relate to *policy*, such as to gather research evidence on the size and spread of the policy problem, generate new insights on what policy solutions had the most positive impact, and encourage greater policy coherence when a combination of policy instruments is essential. It should also inform *policymaking*, such as to learn from experiences of whole-of-government and systems leadership approaches to problems such as COVID-19 ( Cairney, 2025; Cairney & Toomey, 2025). Further, learning should help to close gaps between the provision and use of research evidence for policy, to foster evidence-informed planning for future crises. Otherwise, governments are doomed to make similar mistakes.

The second perspective explains why there is a large and routine gap between these aspirations and reality. The slow and limited responses to COVID-19 inequalities reflect a wider and longer-term pattern in which equity aims are subordinate to higher priorities such as economic growth. Such ‘wicked’ problems endure because inequalities are complex, their definition, importance, and cause is politically contested, and few governments are willing to engage in high state intervention to reduce social and economic inequalities ( Cairney *et al.*, 2021; Cairney *et al.*, 2023; Cairney & Kippin, 2022). The case for greater collaborative policymaking and new forms of systems leadership may be strong in theory but proves to be vague and contested in practice ( Cairney & Toomey, 2024; Cairney & Toomey, 2025). Perfect policy coherence is unattainable, and the pursuit of policymaking integration is contested and a frequent source of unintended consequences ( Cairney, 2025). The pursuit of policy learning and evidence-informed policymaking is a political process for policy actors to navigate, not a technical process for researchers to lead ( Cairney, 2016; Dunlop & Radaelli, 2013; Dunlop & Radaelli, 2018). This contestation extends to policy evaluation, including the measures that we use to determine success ( McConnell, 2010). The ability to transfer success from one territory to another is fraught with difficulties, such as when seeking to identify: the evidence for (and cause of) one government’s success, if an importing/exporting government defines and addresses the same policy problem in the same way, and if their political and policymaking systems are sufficiently comparable ( Cairney *et al.*, 2022a). Therefore, the kinds of policy learning envisaged in the first perspective are not routine features of policymaking.

This comparison of perspectives helps to explain why there is often such a gulf between the huge amounts of research produced on COVID-19 policy (since 2020) and its limited impact or usability for real-world policy and policymaking. On the one hand, scientific research progress - to understand COVID-19 as a virus and ‘design and evaluate vaccine candidates’ – was ‘remarkable’ ( van Schalkwyk & McKee, 2021: iv3-4). On the other, research on non-pharmaceutical interventions (NPIs), such as temporary lockdowns and mask use, was more complex and contested (2021: iv4). Further, most research offering lessons from one country to another proved to be unreliable and vague. Early reviews of research on transferable COVID policy lessons find a patchy literature providing insufficient depth on how and why a government was successful and what could be learned or transferred ( Powell & King-Hill, 2020). Studies generally zoomed into the study of one instrument without relating it to a policy mix or governance dynamics ( Powell, 2023), described key mechanisms vaguely (e.g. strong leadership), and described a ‘whole-of-government’ (WG) approach too generally ( Cairney, 2025). Further, very few studies of policy processes and the COVID-19 crisis focused specifically on inequalities or the inequitable experiences faced by marginalised social groups ( Bromfield & Carey, 2025).

These issues are a particular feature of research on inequalities and their wider economic and social determinants. A wealth of research shows that ‘vulnerable and marginalised’ people will be (1) more susceptible to infection in crowded accommodation, transport, and workplaces, (2) more likely to have underlying health conditions that make them more susceptible to COVD-19 illness and death, and (3) more affected by lockdown measures ( van Schalkwyk & McKee, 2021: iv5-6). Yet, at least initially, few governments collected systematic data on such patterns among social groups, such as in relation to gender, disability, race and ethnicity ( Peci *et al.*, 2021: 7; van Schalkwyk & McKee, 2021: iv5-7). Hence, there was less opportunity to compare policy lessons across countries when the data on inequalities and impact of policies was so incomplete.

We present our review in that real world context. We seek insights from research on how to understand three key components - COVID-19 inequalities, the pursuit of equity, and the potential to generate tangible lessons for policymaking – in a field that struggles to provide usable lessons. It is essential to learn how to inform future crisis responses and anticipate the lack of proportionate and sustained attention to equity, without falling into the trap of assuming that such an important problem will receive proportionate attention or that current research is well equipped to answer our questions. This starting point informs our Methods, such as to (1) set a higher bar on inclusion than in previous reviews, to account for the loose usage of COVID-19 and ‘policy’ without offering clear and transferable lessons for policy and policymaking, and (2) engage in substantial snowballing to find the key texts that engage with policy process research (the final split is 55 included and 30 snowballed).

In Results, we draw on the contrast between aspirational and real-world perspectives to narrate our findings in four parts. First, COVID-19 equity research focuses strongly on gauging the unequal impact of the COVID-19 pandemic and policy. Second, it offers solutions connected to specific inequalities, then expresses some hopes that these policy solutions could be adopted to support more equitable processes and outcomes. Third, however, it highlights a major gap between this aspiration and political reality, such as to document missed opportunities. Fourth, it identifies barriers to the production and use of lessons for future crises, in which there is a disconnect between the processes to generate research on inequalities and its usability and use by policymakers. In the Discussion, we describe this gap identified in COVID-9 equity research - between the size of the problem and the response by most governments – as a much wider problem identified in each of our *Open Research Europe* reviews. The overall message of this literature is that most governments will contribute to very similar inequities in their responses to the next crisis.

## Methods

This is the seventh qualitative systematic review submitted by our team to *Open Research Europe*. To ensure a consistent approach, the following description of Methods draws on the Methods sections of our reviews on inequalities ( Cairney *et al.*, 2021; Cairney *et al.*, 2023; Cairney & Kippin, 2022) and collaborative policymaking ( Cairney & Toomey, 2024; Cairney & Toomey, 2025; Cairney, 2025). Each review adapts Kuckertz and Block’s (2021) guidance on describing systematic reviews with the following categories.

*Rationale*. Our reviews of equity policies are part of the Horizon 2020 project *Integrative Mechanisms for Addressing Spatial Justice and Territorial Inequalities in Europe* (IMAJINE) (Cairney is Co-Investigator). Its aims were to identify how policymakers and researchers understand ‘spatial justice’ and seek to reduce ‘territorial inequalities’. Our role was to examine how ‘(a) policy actors compete to define the policy problem of equity or justice in relation to inequalities’, and (b) ‘identify priorities in relation to factors such as geography, gender, class, race, ethnicity, and disability’ ( Cairney & Kippin, 2022: 5). The project began in 2017 then pivoted to focus on COVID-19 and inequalities from 2020.

*Engagement with previous reviews*. This is our fourth qualitative systematic review focusing on inequalities through the lens of COVID-19 and specific policy sectors (health, education, climate change). The first ( Cairney *et al*., 2021) sought lessons from studies of the use of policy theories in other disciplinary or interdisciplinary fields ( Embrett & Randall, 2014; Munro & Cairney, 2020), and Such *et al*. (2019) guided our first protocol.

*Research/guiding questions*. Each equity policy review has the same guiding questions. Our general focus is:

1. **What is the policy problem?** What is equity, and what constrains or facilitates its progress?
2. **How does it relate to policy processes?** Do articles identify a lack of policy progress and how to address it? What policy theories do they use when describing policymaking?

Each review’s overall guiding question is:

- How does equity research use policy theory to understand policymaking?

The guiding question for article inclusion is:

- How many studies provide a non-trivial reference to policymaking concepts or theories?

Questions to guide analysis include:

- How do these studies describe policymaking?
- What transferable lessons do these studies provide? For example, what lessons for other governments do case studies provide?

Cairney *et al*. ( 2022a; 2022b; 2023) answer those question with reference to inequalities policies relating to territorial politics, health, education, gender mainstreaming, and climate justice. Each review identified some studies of COVID-19 policies as a byproduct of other keyword searches. Here, we search specifically for COVID-19 focused research.

**Figure 1.**
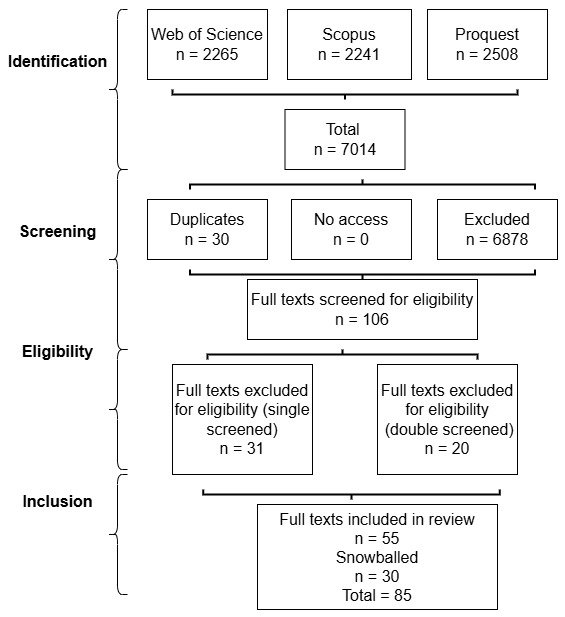
Review process flow chart.

*Databases and initial search terms*. We searched three databases - Scopus, Web of Science, and ProQuest – and adapted our strategy after the Scopus and Web of Science searches. Initially, we used broad search terms – COVID-19 and policy and equity (while shortening the latter terms and using an asterisk search to pick up variants) - to explore the field, but this process became too unwieldy: producing a huge number of results for articles using ‘COVID-19 policy’ as a shorthand without engaging in relevant research or engaging with policy process insights. For pragmatic purposes, we narrowed the search to COVID-19 and policymaking and equity for ProQuest then relied more on snowballing for key texts. We reached a saturation point by the third database search. We learned from previous reviews that the addition of further specialist databases provided minimal additional returns (health) and provided less value than snowballing from the initial set (education, climate). This was a particular issue in this search, which tended to produce texts that discussed COVID-19 and equity but not policy processes, or COVID-19 and policymaking but not equity or inequalities (see Table 1 and Figure 1, summarising search and inclusion results).

**Table 1.** Search results 2024.

Database	Search results	Duplicates	No access	Excluded	Included
Scopus	2241	5	0	2210	26
Web of Science	2265	13	0	2237	15
ProQuest	2508	12	0	2482	14
Grand total	7014	30	0	6929	55

*Note: 0 ‘no access’ for Scopus and Web of Science reflects our use of the ‘Open Access’ filter (which reduced each by 844 and 1757 entries)*.

*Timeliness*. We ran this search from March to September 2024 (last search date was 10/06/24 for Scopus, 10/08/24 Web of Science, 02/09/24 for ProQuest).

*Manual searches and choices regarding initial inclusion*. We used similar criteria for inclusion as the other ORE reviews (including publication in English), but with modest changes to reflect our experience with previous searches and the mass production of COVID-19 research that made searches more unwieldy. First, although we allowed for the inclusion of all relevant published texts (although not pre-prints) – including books and book chapters because our review of education highlighted a relatively high reliance on this format – we also used the ‘Open Access’ filter for the Scopus and Web of Science searches when faced with a mountain of results (we solved this problem in ProQuest by narrowing the keyword search). Second, our previous reviews had used a more manual time-consuming process to exclude most articles if they did not study policy processes. These experiences gave us confidence that a narrower search process – using the term policymaking - would produce similar results. We still use a ‘wide search parameter and low inclusion bar’ ‘to generate a broad narrative of the field, identify a sub-set of the most policy theory-informed articles, and examine how the sub-set enhances that narrative’ ( Cairney & Kippin, 2022: 5; compare with the more restrictive approach in Munro and Cairney, 2020). Third, we included articles that: (1) cite policy theories indirectly via reference to the discipline-specific literature (such as in education that draws on policy sociology) and/or (2) produced relevant work with reference to critical or interpretive approaches to policy discourse or to approaches that would be relevant to our other reviews (such as feminist research), and/or (3) where an insistence on citing mainstream policy theories from the Global North would exclude useful articles produced in the Global South.

Intropido conducted a manual search of the full text to find articles that made at least one reference to an established policy theory (such as multiple streams or the advocacy coalition framework) or concept (such as new institutionalism). Intropido used Cairney (2020) for a list of mainstream theories and concepts, which are also summarised on Cairney’s blog. Cairney performed a further inclusion check while analysing each article, referring some back to double check for exclusion. Cairney and Intropido double-screened and excluded 20 texts during the final eligibility phase (using full-text analysis). However, we cite 16 of the 20, largely for readers seeking more on COVID-19 inequalities in texts that do not explore policy processes in sufficient depth for our review ( Abel & Siraj, 2024; Adams *et al.*, 2023; Addo *et al.*, 2022; Adeola, 2021; Adigun, 2024; Agbelie, 2023; Aggarwal *et al.*, 2023a; Aggarwal *et al.*, 2023b; Aguirre *et al.*, 2021; Aiona *et al.*, 2022; Ajibola *et al.*, 2022; Akbarali *et al.*, 2023: 1; Akintobi *et al.*, 2020; Akintunde *et al.*, 2023; Akobirshoev *et al.*, 2022; Baker & Sorensen, 2024). This list also exhibits the initial search process uncertainty (most are bunched in A-authors during a process of initial learning).

*Routine data collection*. We coded the following aspects of each included article (in Excel):

- *Country/ region of study*. 29 (52.7%) of the 55 included texts focused on single-country contexts, including: 9 USA, 6 UK, 3 Canada, 2 Iran, South Korea, and 1 of Israel, Italy, Mali, Myanmar, Peru, the Philippines, South Africa. 17 studies (30.9%) adopted a global perspective, examining cross-national trends or international policy frameworks. 6 (10.9%) provided a regional outlook, focusing on specific areas such as Africa, the Americas, East Asia, Eastern Europe, the Global South, and the Middle East & North Africa. 3 (5.5%) analysed multi-country contexts that transcend regional boundaries, covering Australia and Canada, Australia, Canada and New Zealand, and Denmark, India, El Salvador, Brazil, and the EU.
- *Country of author affiliation*. Of the 55 authorial teams, 49 feature scholars from English-speaking countries (18 US, 17 UK, 5 Canada, 4 Australia, 1 New Zealand), and European institutions (2 from Denmark, Finland, France, Italy, Netherlands, and 1 from Bulgaria, Germany, Hungary, Ireland, Moldova, Romania, and Spain). 5 teams include MENA-based scholars (3 Iran, 1 from Algeria, Israel, Kuwait, Morocco, Qatar, Saudi Arabia). 3 teams feature African institutions (South Africa, Botswana, Mali, Senegal) and 3 Asian institutions (India, Philippines, South Korea).
- *Policy or case study issue*. 13 (23.6%) studies examine health equity and inequalities affecting marginalized communities. 7 (12.7%) studies examine education policies (schooling, assessment, equity in learning). 6 (10.9%) examine the legitimacy of experts and public trust in policy processes. 5 (10%) study emergency responses and public health interventions, plus 4 (7.3%) on vaccine equity, 3 (5.5%) social security, 3 food, 2 (3.6%) waste management, 2 urban knowledge/local policy design, 2 health evidence policymaking, 2 global governance (the rest were individual studies).
- *Research methods*. 31 (56.4%) qualitative approaches, including case studies, interviews, discourse analysis, and ethnographic methods. 14 (25.5%) literature reviews. 6 (10.9%) mixed-methods research (qualitative and quantitative). 4 quantitative (statistical modelling, surveys, or large-scale data analysis.
- *Article or book type*. 43 (78.2%) were research articles, 7 (10.9%) journal commentaries, 3 book or book chapters (5.5%). 1 (1.8%) brief report, 1 research protocol.

We also gathered general information in relation to three guiding questions ( Cairney & Kippin, 2022: 7):

1. How do the authors define concepts such as inequalities and equity? (summarised in Results ‘The impact of COVID-19 reflected and reinforced inequalities’)
2. What, if any, are their policy recommendations? (summarised in Results ‘COVID-19 prompted many solutions for change’)
3. On what policy concepts and theories do they draw? We find that COVID-19 research generally fulfils two of the three concepts (COVID-19, equity, policymaking). Hence, a very small subset of included research provides detailed references to public policy theories, which we supplement by snowballing from key sources (e.g. Naem *et al.*, 2023). Further, most COVID-19 research exploring policy processes tends to mention equity superficially.

*Data analysis, aggregation, and presentation*. One author (Cairney) used an inductive qualitative approach to analyse each text, generate themes (Results), and relate them to previous reviews (Discussion). As Cairney and Kippin (2022) note, ‘the rules associated with this method are less prescriptive than with its quantitative equivalent, suggesting that we (a) describe each key judgement, and (b) foster respect for each author’s methods and aims’ (see Sandelowski & Barroso, 2007: xv). The 20000-word limit allows us to perform the latter. In a separate Word document, Cairney produced summaries of each text’s story (including research aims and question, approach, findings). Cairney condensed these summaries to produce thematic findings from the studies as a whole, and used a sub-set of relatively theory-informed texts to describe the results of engagement with policy concepts. We did not perform any quantitative tests to assess risk of bias. We present a narrative systematic review (rather than qualitative coding aided by tests for inter-coder reliability). We use general descriptions of ‘most’ or ‘few’ texts to aid that narrative, not signal precise proportions of the included texts.

### Limitations relevant to data gathering and analysis

Previous reviews note limitations regarding the loose usage and minimal definition of key terms, which – in this review – includes a general focus on policy learning or lessons without sufficient detail ( Powell, 2023). Further, studies engage sparingly with policy theories and concepts when describing policy processes. In that context, while it is possible that a more time-consuming manual search for research on *policy* rather than *policymaking* would have yielded further results, we do not consider it an efficient use of review time. We respond by keeping the bar for inclusion low to encourage immersion in the field, then snowballing to identify key reference points. As with each of our reviews, the starker limitation relates to the bias in research towards Global North researchers and experiences. We did not restrict our search geographically, but the requirement for publication in English strongly influences the results. There is some spread of countries in this review, but the list of included texts is not representative of a global experience. Therefore, the Results and Discussions sections have clear implications for policy and policymaking, but their application is not universal.

The complete search protocol, PRISMA checklist, and structured bibliography ( Cairney & Intropido, 2025) is stored on the OSF https://osf.io/ue36c/ ( Cairney & Intropido, 2025).

## Results

We present a four-part Results narrative of COVID-19 equity research. This research (1) identifies the unequal impact of the COVID-19 pandemic and policy, then (2) expresses some hopes for policy solutions to support more equitable processes and outcomes. However, it (3) highlights a major gap between aspiration and reality (or the proposal versus acceptance of such solutions), and (4) identifies barriers to the production and use of lessons for future crises.

### 1. The impact of COVID-19 reflected and reinforced inequalities

The most general and frequently-expressed finding is that the COVID-19 pandemic – and most government ‘lockdown’ responses - reflected and reinforced health inequalities:

‘COVID-19 is a syndemic of infectious disease and inequalities. It has killed unequally, been experienced unequally and will impoverish unequally. These health inequalities, before, during and after the pandemic are a political choice – with governments effectively choosing who gets to live and who gets to die’ ( Bambra *et al.*, 2021: xiv; see also Green *et al.*, 2021: 10–11; Bambra *et al.*, 2020)

Bambra *et al.* (2021: xiii-xiv) describe a collection of mutually-exacerbating inequalities that were reinforced by COVID-19, including

- *Unequal infection rates and deaths* among poorer and ethnic minority populations, reflecting ‘existing social, economic and health inequalities’ (2021: xiii), such as when ‘deprivation’ and ‘structural discrimination’ contributed to ‘increased exposure to the virus’ and less access to ‘culturally appropriate methods’ to share information and offer services ( McKee *et al.*, 2022: 241).
- *Unequal impacts of the COVID-19 lockdown policies* that closed most public places, thus restricting the ability to access education, work, or maintain economic activity. Emerging patterns showed ‘low-income workers, women and ethnic minorities bearing the brunt’ ( Bambra *et al.*, 2021: 42–45). These impacts included the exacerbation of poverty and low income from job losses, the higher exposure to infection – among some racial or ethnic groups - via public-facing key worker jobs, and a shift to online education to which only some students could pivot. Further, lockdowns made women and girls more vulnerable to domestic violence and sexual assault, and school closures and illness among older people affected women disproportionately since they were more likely to take on more caring responsibilities (see also Adeola, 2021; Fortier, 2020; Wenham *et al.*, 2020)
- *Political choices with unequal effects*. Before COVID-19, public health researchers already emphasised a lack of government attention or response to the unequal spread of non-communicable diseases (NCDs) - such as cardiovascular and respiratory diseases, cancers, and diabetes – in relation to factors such as income, race and ethnicity, disability, and gender ( Theis & White, 2021). During the pandemic, it became clear that this unequal spread of NCDs contributed to the pre-existing conditions that increased susceptibility to COVID-19 harm. Hence, both the unequal spread of NCDs and COVID-19 impact highlight the profound contribution of social and economic factors to ill-health, *and* the choice by most governments to not fully mitigate health and other inequalities via direct payments (such as social security and employment protection) or funding for essential public services.

These patterns of unequal outcomes contrast with policymaker narratives about values of social solidarity and everyone being in it together. Rather, Bambra *et al.* (2021: 8–9) describe unequal (1) ‘vulnerability’ and (2) ‘susceptibility’, since NCDs and ‘long term exposures to adverse living and environmental conditions’ make people more vulnerable to COVID-19 illness and death; (3) ‘exposure’, since lower-paid workers in service jobs are less able to work safely from home; and (4) ‘transmission’, since more deprived neighbourhoods have smaller homes shared by more people (see also Ahen, 2021; Davis, 2022). These dynamics intersected with the more visible impact of COVID-19 in relation to age, including examples of hospital rules to limit scare healthcare resources in relation to age and co-morbidity, and unchallenged social norms that led to younger people not taking seriously the risks of their COVID-19 behaviour to older people (described by McDonald, 2022: 251 as ageism and ‘lethal neglect’). The pandemic also had a profound impact on global inequalities, such as to undermine or reverse progress in meeting the UN Sustainable Development Goals ( Saguin & Howlett, 2022: 2).

Multiple studies contribute to this general finding of inequitable policy outcomes and processes, including:

***The mistreatment of migrant workers***

Istiko *et al.*’s (2022) scoping review (Australia, Canada, and New Zealand) finds widespread inequity regarding the treatment of migrant workers during COVID-19. Discrimination related to physical distancing restrictions that kept them in cramped and unsafe accommodation, threats of deportation by employers, and poor access to health services and social security: ‘exploitative employment practices, precarity, and racism contribute to the continued exclusion of temporary migrant workers’ (2022: 1).

***Marginalisation by race and ethnicity***

Akintobi *et al.* (2020: 1) show that, ‘African Americans are more likely to contract … COVID-19, be hospitalized for it, and die of the disease when compared with other racial/ethnic groups’. These inequalities relate partly to pre-existing health conditions and unequal access to healthcare (2020: 1). They also reflect multiple social determinants of health relating to historic segregation into lower income neighbourhoods with less access to healthy places and food, and less representation in relation to the making and delivering of policy (including within the medical profession making choices on treatment and resource allocation – Wright and Merritt, 2020: 821–2). Naem *et al.* (2023) identify the impact of systematic racism on health inequalities, which exacerbated and were exacerbated by COVID-19, including direct oppression in relation to citizenship, unequal ability to engage in healthy behaviour, unequal access to public services, and the stress and other impacts on health caused by discrimination (2023: 835; Amri & Drummond, 2021). Further, ‘the pandemic exacerbated existing high levels of malnourishment and disparities in food insecurity based on race, ethnicity, and nationality’ ( Ilieva *et al.*, 2023: 20).

***The marginalisation of minority voices***

Poor democratic processes exacerbate this marginalisation. Some social groups are disproportionately harmed by COVID-19 *and* have little voice in policy processes focused on mitigating harm:

“Whilst governments worldwide are claiming that their responses are ‘responsive and equitable’, it is reasonable to expect that the impacts of COVID-19 will turn out to be highly inequitable. Those who we might expect to be disproportionately impacted will be those whose voices are muted in policy processes; in past crises, these were the poor, women, racialised minorities, and other disadvantaged groups, both at home and abroad” ( Henson *et al.*, 2020: 1340–41).

Many governments relied unusually highly on elite scientific expertise over lay knowledge, contributing to the formulation of policies based on social settings deemed (inappropriately) to be typical. For example, physical distancing policy may be based on assumptions of a typical home or public sphere, to the detriment of ‘people living in high-density informal settlements’ ( Henson *et al.*, 2020: 1347, citing Onditi *et al.*, 2020).

Multiple studies link such unequal treatment or outcomes to specific sectors, including:

***Limited access to essential public services such as health and social care***

Bascolo *et al.* (2022: S615) describe problems with the COVID-19 response in South American countries, linked to fragmented services and limited access. Similarly, Ponce de Leon (2021) describes a pandemic exposing the insufficient capacity of – and access to – healthcare in South America, with a greater impact on groups unable to afford health insurance. Further, multiple studies track the adverse impact of a shift to online modes of health or social care services for disabled and ‘socially vulnerable populations’ ( Adams *et al.*, 2023; Agbelie, 2023; Akobirshoev *et al.*, 2022; Baker & Sorensen, 2024; Davis, 2022).

***Unequal vaccination access***

Multiple country studies describe the unequal access to, and uptake of, vaccines in relation to marginalised social groups, prompting a need to understand solutions such as tailored communication or to boost trust in local or community leaders when trust in government is low ( Aggarwal *et al.*, 2023a; Ajibola *et al.*, 2022; Akintunde *et al.*, 2023; Cohen & Levavi, 2023). Within countries, such problems may be exacerbated by policies connected to Covid vaccination or immunity, such as when some social groups have less access to vaccination or testing to demonstrate they are post-infection, or when policies explicitly favour ‘more privileged populations’ ( Dada *et al.*, 2021: 7). Global studies also highlight the profoundly unequal distribution of vaccines ( Kavanagh & Singh, 2023: 233–5), often despite the rhetoric of international cooperation ( Mudaly & Chirikure, 2023: 3).

***Education inequality***

The impact of school or education closures, and a shift to online learning, exacerbated educational inequalities such as among low-income populations with less access to IT and data, or less time to devote to caring responsibilities: ‘the crisis was not equally distributed: the most disadvantaged children and youth had the worst access to schooling, highest dropout rates, and the largest learning deficits’ (Arar *et al.*, 2024; Kim *et al.*, 2021; Mitescu-Manea *et al.*, 2021; World Bank, 2020: 5; see also Cairney & Kippin, 2022). Government policies may also exacerbate inequalities when using blunt tools to moderate assessment of attainment ( Carter & McIntee, 2021: 234; Kippin & Cairney, 2022a; Kippin & Cairney, 2022b) or not anticipating limited access to a bank account for emergency financial assistance ( Morgan *et al.*, 2021: 1).

***Financial stress***

An *et al.* (2023: 1300) find that governmental restrictions on mobility (lockdowns and travel bans) were economically regressive. They have major and unequal effects on household financial stress, especially when social security ‘safety nets’ are low and households already face poverty (which skews towards younger households). The economic impact of COVID-19 and lockdowns also, ‘threatened the survival of many rural livelihoods and small medium and micro enterprises (SMMEs) across the globe’ ( Farisani, 2022: 1).

### 2. COVID-19 prompted many solutions for change and some hopes for greater state intervention

Equity research emphasises the urgent need to learn lessons to help improve policy responses to crisis: ‘We need to learn from COVID-19 quickly to prevent inequality growing and to reduce health inequalities in the future’ ( Bambra *et al.*, 2021: xiv).

***COVID-19 research has produced a long list of proposed solutions***

There is no shortage of such solutions, and most emerge directly from the definition of the problem. For example, Bascolo *et al.* (2022: S615) describe problems with the COVID-19 response in South America, linked to ‘structural deficiencies of health systems in the Region, including segmentation, limited resources, and the fragmented organization and delivery of health services, all of which are aggravated by high levels of informal labor and social inequities’. Hence, solutions focus on: giving greater priority to health equity in relation to social and economic policies (including social protection policies), an integrated system of health and public services focused on proper surveillance, universal coverage, and addressing social determinants, and reducing inequalities in health and access to care (2022, citing Etienne *et al.*, 2020). This problem-solution approach is a general feature of research seeking:

- More equitable healthcare access ( Adigun, 2024; Agbelie, 2023; Aissaoui *et al.*, 2022; Akbarali *et al.*, 2023: 1), including telemedicine ( Adams *et al.*, 2023).
- Tailored vaccination policies to improve trust and communication among marginalised groups and address their lower priority during policy delivery ( Addo *et al.*, 2022; Aggarwal *et al.*, 2023a: 4; Aggarwal *et al.*, 2023b; Aiona *et al.*, 2022; Ajibola *et al.*, 2022; Cohen & Levavi, 2023; see also Kalajahi *et al.*, 2022).
- Packages of measures to redress inequitable vulnerability to COVID-19 harms among African American populations, such as funding for essential non-profit community organisations and health workers, food and essentials vouchers, and online access for education and work ( Akintobi *et al.*, 2020: 6; Wright & Merritt, 2020: 822–4).
- Food policies to reduce nutrition inequalities and boost support for low-paid food workers ( Ilieva *et al.*, 2023: 21).
- Support for small rural businesses ( Farisani, 2022).
- Improved models for regional lockdowns ( Abel & Siraj, 2024).

***This research calls for greater state intervention to boost public and economic health***

Generally, these studies coalesce around the argument that much higher state intervention is essential, such as to provide greater health protection (and childcare) for essential workers, the right to paid sick leave, social security and employment support during lockdown, universal healthcare (including mental health services, such as to address higher isolation), adequate public transport to access healthcare, and services to provide access to nutritious food (e.g. Bambra *et al.*, 2021: 105–7, citing Jones *et al.*, 2020: 1853; Bhaskar *et al.*, 2020; Goldblatt *et al.*, 2020: 39–43; see also Gaynor and Wilson, 2020: 836).

This push for reform comes with reference to evidence of the mutually reinforcing value of high state intervention for public health and economic policies: ‘Economists and public health experts have argued that policies to protect public health are the only way to restart the economy’ ( Warner & Zhang, 2021: 186). For example, this intervention often relates to income support and social security measures, such as to show that US states with a substantive ‘social safety net’ were able to lock down more quickly and face less pressure to reopen too early, compared to states who reopened earlier and ‘began experiencing severe spikes in COVID-19 infection rates’ (2021: 186). Such evidence reinforces conclusions from previous eras in which bursts of state intervention reduced health inequalities considerably (see Bambra *et al.*, 2021 on German reunification from the 1990s and UK reforms from 1997 to the early 2000s).

***Some identify the need to prepare more effectively for the impact of lockdowns***

Studies of education inequalities highlight the adverse and unequal consequences of lockdown policies on education provision and attainment (see Cairney & Kippin, 2022). A common theme is that country or regional governments acted quickly to close schools in 2020 and move education online, without developing specialist capacity (teachers and IT) to foster online learning. Although such initial action was understandable in a context of high uncertainty, it now highlights the need to plan for future crisis by building resilient capacity and anticipating the unequal impacts of a crisis and crisis response. Examples include:

- Arar *et al.* (2024) examine COVID-19 responses in Kuwait, Lebanon, Morocco, Palestine, and Qatar. Their lockdown policies ‘were not as inclusive as they should be because they benefited the high and middle income populations but left behind the poverty-stricken pupils’ (2024: 132). They recommend a more decentralised policy process to adapt to educational inequalities while the centre responds to the primary crisis (2024: 134, citing van der Wal, 2020 on empowering public managers).
- Carter and McIntee (2021: 234) describe the inequitable effect of a shift to online education in relation to ‘learning of Black, Indigenous and students of color’, exacerbated by US government a fixation with test scores to rank people and schools. Apart from rejecting the ‘colonial logic’ of neoliberal approaches to education, they recommend that the federal government funds public high-speed internet to address inequality of access to information (see also Chaufan *et al.*, 2023 on Canada).
- Mitescu-Manea *et al.* (2021: 546) examine how international organisation, NGO, and expert reports conceptualised the impact of COVID-19 on educational inequity in Romania, Hungary, Bulgaria, and Moldova. Organisations highlighted the inequitable impact of school closures and unequal access to online learning (‘digital poverty’), then proposed short-term support to address urgent issues - such as pre-school and early primary students not gaining any benefit online, the inequalities of exam preparation for high school students, and cyberbullying - and longer-term reforms to address chronic inequalities (2021: 550). There was also some hope of harnessing bursts of social solidarity boosted by ‘grassroots action, organized civil society and corporate philanthropy’, who ‘stepped in with concrete actions where they felt that governments were failing the most vulnerable’ (2021: 544).

***This research comes with modest optimism for change, qualified by experience***

Such examples highlight the modest optimism that the COVID crisis *might* spark positive changes to policy and policymaking (such as via new approaches to policy design, Salinas, 2022). In other words, perhaps greater intervention could be more politically feasible now than before, especially in ‘neoliberal’ states where governments were previously reluctant to intervene. For example, Belon *et al.* ( 2024: 9, citing Glenn & Nykiforuk, 2020) describe an opportunity to learn from the ‘unexpected, but positive, consequence of temporary actions for equity-deserving populations’, such as the ‘less punitive work benefit programs, expansion of paid sick leave, free public transportation, and wage top-up for low-income workers’ which helped to reduce ‘housing affordability stress’ and boost ‘social solidarity’. More generally, An *et al.* (2023: 1300; 1313) identify the need to address social equity when introducing emergency financial provisions to mitigate income loss, such as to favour more targeted wage support than general cash transfers.

Regardless of researcher optimism, there is a strongly and frequently expressed belief that this new experience *should* focus the minds of policymakers:

‘Building a better future from COVID 19 requires policies and interventions that reduce inequities, address vulnerabilities first and leave no one behind’ ( Goldblatt *et al.*, 2020: iv).

The alternation, between a language of (1) hope for new and ambitious solutions, and (2) exhortation to do better, is recurrent in this literature. Researchers narrate what they think *could* and *should* happen, but almost always accompanied by more realistic expectations – of what *will* happen - based on experience. For example, the idea of a ‘window of opportunity’ for change is expressed frequently, including Bambra *et al.* (2021: 109–19) on opportunities for new models of economic growth and social protection, political challenges to economic elites and democratic innovations more generally, renewed trust in science, and lessons from COVID-19 for other crises such as climate change:

‘Clearly, the Covid-19 crisis offers a window of opportunities for change toward more sustainability. It might trigger or speed up changes that otherwise wouldn’t have come so quickly, or make room for suggestions and decisions that promote a more sustainable way of looking at globalization, global urbanization, biodiversity protection and climate change mitigation’ ( Haase, 2020: 5, cited by Bambra *et al.*, 2021).

Similarly, Naem *et al.* (2023: 835) describe COVID-19 as a potential driver of a window of opportunity to new ideas and ways of working associated with World Health Organisation Health in All Policies (HiAP – see our Discussion section) or Healthy Cities agendas, such as to harness ‘cross-sectoral collaboration, community participation, political commitment’:

‘It is essential to take action to support addressing health inequities within cities, which is particularly timely given the recent COVID-19 pandemic, which mobilized governments and has made health a salient issue that can be capitalized on to address the social determinants of health’ ( Naem *et al.*, 2023: 835).

Further, a series of snowballed articles (from Naem *et al.*) use policy theories to help describe this aspiration for changes to policy and policymaking. First, Amri and Logan (2021: 1190–93) use the multiple streams framework ( Herweg *et al.*, 2023; Kingdon, 1984) to identify:

1. “An opportunity for the framing of inequity and imbalanced SDH [social determinants of health] as a ‘problem’ that needs to be addressed”,
2. some hope that the pandemic experience would boost the political feasibility of expensive state interventions to protect population health, and
3. a ‘shift in national moods towards overcoming inequalities’ even when NPIs are restrictive, including social/physical distancing and mask use regulations.

On the other hand, such vague aspirations do not translate into action when ‘many governments realize the importance of the SDH … [but] do not seem to grasp the importance of making the necessary financial and political investment’ (2021: 1193).

Second, Amri and Drummond (2021) use the language of punctuated equilibrium theory ( Baumgartner & Jones, 1993; Baumgartner *et al.*, 2023) to describe the rapid and profound impact of COVID-19 on policy and policymaking, which should help to challenge the idea that governments cannot respond quickly to major policy problems. Changes include a shift in policy framing, inclusion of new actors in networks, and processing policy in new venues ( Amri & Drummond, 2021: 37–9). They argue for governments to harness this energy for change, which includes boosting the political feasibility of policies to compensate people for the disproportionate economic impact of COVID-19, such as addressing gig workers’ loss of income, and subsidised daycare to address a loss of school support for women. However, this argument is mostly exhortation rather than based on demonstrable and enduring progress:

‘It would seem we no longer need to accept the excuse that problems are too difficult or will take too long to tackle. The pandemic demonstrated that government can act boldly and with lightning speed when immersed in something perceived as a crisis. However, governments’ records of learning lessons from past crises are not strong’ (2021: 39).

Third, Amri *et al.* (2022) perform a review of reviews (10 studies relating to HiAP and other strategies) to identify the enablers and barriers to intersectoral action (a concept central to HiAP and other public health narratives - see Cairney *et al.*, 2021; Cairney, 2025):

‘Enablers detailed are: (1) systems for liaising and engaged communication; (2) political leadership; (3) shared vision or common goals (win-win strategies); (4) education and access to information; and (5) funding … These findings provide a rigorous evidence base for policymakers to inform intersectoral and multisectoral approaches … to work towards health equity’ ( Amri *et al.*, 2022: 1).

However, they also conclude that there is minimal evidence for the tangible benefits of such action, as well as very low attention to equity in such initiatives (2022: 9).

***Modest expectations come with policy ambiguity***

These hopes for policy change come with some uncertainty about the meaning of equity among key advocates (e.g. Adigun, 2024). For example, Amri *et al.* (2021: 1) present a scoping review on the WHO approach to health equity, arguing that it ‘has held - and continues to hold - ambiguous, inadequate, and contradictory views of equity that are rooted in different theories of social justice’. On the one hand, the WHO publicises Whitehead’s (1990: 5) definition of inequity - “differences which are unnecessary and avoidable, but in addition, are considered unfair and unjust” - and has moved on from merely measuring inequalities to making a social justice case for equity (2021: 1–2). On the other hand, various WHO documents and strategies do not live up to this definition, partly because it is highly ambiguous and the ambiguity is never resolved (what exactly is unfair? 2021: 2; 10). Sometimes, the WHO has focused on individuals when engaged in measurement or social group differences to emphasise the social justice case. At other times, it has used a low baseline of health for all (akin to the OECD emphasis on a baseline of education attainment) *and* a strong emphasis on reducing health inequalities in relation to social and economic inequalities (2021: 11–13). Amri *et al.* (2021: 14) argue that the time for Whitehead’s definition to provoke debate has passed, and that the WHO should clarify that it equates health equity to ‘Sen’s Capabilities Approach’ which emphasises the unequal ability of people to act to boost their own health (such as when they have fewer resources or face more discrimination).

Similarly, Campbell *et al.* (2023: 170) describe a lack of policymaker attention to health equity, relating not only to ambiguity (‘the definition of *health equity* is not universally agreed’) but also the power to define equity, from a narrow focus on access to healthcare or broad focus on social justice (2023: 171). They explore strategies for raising health equity on the agenda enough to promote substantive action. For example, in Myanmar, the context relates to a military regime overseeing command-and-control policymaking, facing international sanctions and internal conflict, and possessing limited funds for change. The low priority of equity combines with its lack of visibility (e.g. relating to limited data), plus competition to define equity in terms of access to health services or wider measures such as life expectancy. Here, there is some scope for strategic engagement to sell a policy to policymakers sceptical of preferred reforms (their example is to propose politically feasible voucher schemes rather than more technically feasible universal health insurance - 2023: 174–6).

Capper *et al.* (2023: 4–5) find high levels of ambiguity in UK government policy documents for England: there is a tendency to provide vague references to wider determinants of health without actionable policies for named organisations (it is everyone’s and therefore no-one’s problem) while focusing only on the most-at-risk or emphasising ‘lifestyle’ advice. Hence, meaningful policy progress requires a clear definition of the wider determinants of health accompanied by clear responsibilities among empowered organisations (e.g. in local government and communities). It also requires a ‘positive vision for a society with equitable health’ and ‘proportional universalism’, which describes a whole population focus which allows for targeted action (2023: 5–6).

In the US context, Gaynor and Wilson (2020: 836) describe ‘targeted universalism’: ‘a framework to develop inclusive policies and programs that consider the needs of all groups in order to move everyone toward a universal goal’. Their five-step process is to: identify ‘a universal goal stemming from a shared societal problem’, relate it to differential progress among social groups and identify which groups are below the ‘baseline metric’, then ‘understand how existing structures advance or limit vulnerable populations from achieving the universal goal’ to allow policymakers to respond with financial and public service support (2020: 836). Here, crystal-clear framing is essential to constitutional and policy issues, since the statement of a universal goal reduces the risk of legal challenge to targeted programmes (2020: 836). Clarke (2023) explains this need to address potential conflicts between US constitutional law versus health equity approaches in public policy. The former focuses on overt and provable discrimination and rejects statistics on disparities as evidence of intentional harm. The latter focuses on direct and indirect forms of individual and structural causes of health inequalities. Hence, while public policies may seek to redress the unequal impacts of COVID-19 and COVID-19 policy, they may be rejected in the courts on the grounds of favouring one social group with policy (even when seeking to get round this threat by emphasising a place-based rather than population-based approach). Hence, clarity and imagination are required to address disparities without facing legal challenge, such as to fund activities – including community outreach and communication campaigns – that have universal coverage but also greater benefits for some social groups.

***Modest expectations come with governance uncertainty***

Hopes for reform come also come with uncertainty and debate about the best governance model to support progress. For example, Koon *et al.* (2021: 1) conclude that regulations/mandates are essential in the US, since only providing guidance or appealing to voluntarism would not be effective with people who see COVID-19 as a conspiracy or behavioural change as a threat to their livelihood. However, Becher *et al.* ( 2021, cited by Abbasi *et al.*, 2023) find that this association between ideology and compliance is not a feature of most countries, which allows for different approaches. For example, Abbasi *et al.* (2023: 27–8) argue against simply using top-down measures to ensure COVID-19 compliance among waste pickers in Iran, since it may exacerbate low levels of trust in government and science. Instead, more bottom-up and collaborative measures - involving community leaders, and increasing government transparency - would fuel trust-based behavioural change, aided by social protection funding to allow waste pickers to not work during lockdowns (see also Cohen & Levavi, 2023; Farisani, 2022). Similarly, Acuto *et al.* (2021: 105295) describe the potential to foster bottom-up approaches to evidence-informed policymaking, such as to combine diverse sources of knowledge and give voice to marginalised actors, foster trust in well-established knowledge hubs (‘urban observatories’), and monitor the unequal effects of market and political choices ‘supporting sustainable urban development’ (2021: 105301 in Johannesburg, Karachi, Freetown and Bangalore).

Such examples highlight a variety of ideas on how to foster improved governance to promote health equity. Many emphasise the vague notion of taking a whole of government (WG) and ‘whole of society’ approach to health ( Green *et al.*, 2021: 10, citing Rogerson *et al.*, 2020) or a ‘One Health approach’ to preventing zoonotic transmission among the wildlife trade ( Aguirre *et al.*, 2021; see Cairney, 2025 on the popularity of a vague WG language). Some seek a more meaningful redistribution of global political power. For example, Kheir-Mataria *et al.*’s (2023: 4) Delphi panel describe facilitators of equity as ‘better representation of countries from the global south’, ‘a mechanism to monitor the influence of private actors and non-governmental financing organizations in policymaking’ (to address vaccine profits and inequitable access), ‘change the role of the World Health Organization’ and foster more powerful and effective global health governance and accountability. Similarly, Kavanagh and Singh (2023) describe a binding international agreement, backed by an authoritative international organisation, as essential to policies such as the equitable distribution of vaccines.

Others focus on using more effective models of implementation science to reduce the unequal benefits of schemes designed to mitigate inequalities: ‘Inequities in implementation contribute to the unequal benefit of health interventions between groups of people with differing levels of advantage in society’ ( Gustafson *et al.*, 2023; cited by Nitto *et al.*, 2024). For example, Nitto *et al.* (2024: 1) recommend more flexible payments to reduce administrative burdens while implementing the ‘Special Supplemental Nutrition Program for Women, Infants, and Children (WIC)’ provided to ‘over 6.2 million pregnant, postpartum, and breastfeeding women and children up to age five’ in the US in 2022.

### 3. The gap between aspiration and reality: windows of opportunity to exacerbate inequalities

COVID-19 equity research tends to highlight a profound gap between policy aspirations and policymaking reality. If there is a window of opportunity for policy change, it tends to open and close too quickly to produce politically feasible solutions, or it produces changes that exacerbate inequalities.

***Resolving ambiguity in favour of individual over state responsibility***

Policy ambiguity tends to be resolved in favour of individualist or market solutions rather than state intervention (see this theme in HiAP research, Cairney *et al.*, 2021). For example, Theis and White (2021) describe a clear impetus to reform UK government obesity policy for England, since the pandemic demonstrated greater ‘risks for people living with obesity, such as an increased risk of testing positive for COVID-19 and of hospitalization, as well as advanced levels of treatments and death’ (2021: 128). However, their analysis of three decades of policy shows that the UK government is reluctant to shift from giving guidance to individuals on their lifestyle choices towards a social-determinants focus on population measures, such as state intervention to regulate industry products and advertising, and tax unhealthy foods or limits sales, while making healthy food more affordable and accessible (2021: 127). Further, UK governments design policies that are not amenable to implementation and ‘rarely commission evaluations of previous government strategies or learn from policy failures’ (2021: 127).

Studies engaging directly with policymaker and practitioner perspectives on COVID-19 find a similar absence of social determinants lenses and a general sense that ‘health inequity is rarely considered in the design and evaluation of public health interventions’ ( Gagnon-Dufresne *et al.*, 2022: 10). Boivin *et al.* (2023) argue that WHO COVID-19 guidance makes a vague reference to health equity, contributing to minimal policy clarity. In their case study of Mali, policymakers related equity to free testing, then reactive visits, and a phone number to record infection. Gagnon-Dufresne *et al.* (2022) examine the extent to which inequalities were considered in planning for vaccination delivery in Montreal. They find, among practitioners, high attention to the social determinants concept but variability in interpretation coupled with a lack of data on racialised populations. The crisis response began as whole population approach (e.g. for testing) followed by gradual increases in ‘accessibility for specific population groups, addressing information, linguistic, physical, and geographic obstacles to COVID-19 testing’ (2022: 7).

Further, Ilieva *et al.* (2023: 17–18) describe limited attention to issues such as ‘gender equity in urban food policy’ as well as ‘missed opportunities’ for more radical or equitable ‘upstream’ food system policy changes, such as redistributive tax and social security measures, greater support for ‘immigrant food workers’, or an expansion of opportunities for businesses owned by minority or women entrepreneurs’.

***Policymakers learn lessons through the lens of their beliefs***

Researchers routinely provide lessons from comparisons of countries, but the audience may not be receptive to the conclusions. For example, Belon *et al.* (2024: 8) describe Canadian financial assistance policies (2020) as ‘more progressive and equity-focused than comparable policies in Australia’ but relate the difference largely to left versus right wing parties of government and a more ‘neoliberal’ approach in Australia. As such, it is not clear how or why Australian governments would seek to learn from Canada, and what lessons would be available on the impact of Canadian funding, especially if such funding relates to a value judgement on distributing resources rather than a wider population payoff.

Similarly, while Warner and Zhang (2021: 186) argue that US states with a ‘social safety net’ were able to lock down more quickly and face less pressure to reopen too early, the main determinant of policy was often the party in charge:

‘Public health has become politicized in the US. States with unified Republican control imposed shutdown orders later and reopening orders sooner, despite their level of COVID-19 infections or demographic characteristics related to higher infection rates. The politicization was influenced from the top, as President Trump expressed impatience with public health directives and focused more on the need to open up the economy. Consolidated partisan control across legislative and executive branches led Republican governors in Republican-controlled state legislatures to follow the president’s lead and open up’ (2021: 186).

***Making but not delivering on vague commitments***

Studies find limited incentives for elected politicians to make or implement concrete commitments to reduce inequalities. Some elected (US) politicians seem to incentivised to delegate key choices to bureaucracies, thus fudging accountability ( Curley *et al.*, 2023). Or, they support modest or temporary commitments towards domestic policy change. For example, Maino and De Tommaso (2022) describe the window of opportunity for anti-poverty policies in Italy, prompted partly by the trial of a minimum income scheme (Citizenship Income) and the need for greater financial assistance during COVID-19. However, the scheme was too limited in scope during the pandemic (e.g. disadvantaging large families and migrants), prompting additional temporary emergency assistance for new households in poverty). Although COVID-19 outcomes exposed the issue of poverty, the CI scheme was not sufficiently boosted to become a structural solution, largely because it was opposed by too many centre-right political parties.

Other policymakers embody incentives to make vague global commitments while protecting domestic interests. For example, Kavanagh and Singh (2023) describe a failure of ‘international mechanisms’ to ‘achieve equitable global distribution of COVID-19 vaccines’. Problems included the lack of a legally binding commitment to distribute vaccines in relation to population size. Rather, richer countries agreed to voluntary measures, but hoarded stocks for their own populations (‘vaccine nationalism’), objected to technology-sharing and production in low-and-medium-income-countries (LMICs), and provided modest support for global distribution (2023: 233–5).

***Policy windows for exacerbating inequalities***

Other studies suggest that COVID-19 policies prompted policy windows for the exacerbation of inequalities, such as when the initial window prompted the prioritisation of safety via lockdown then:

- treating education equity as an ‘agenda for future reform’ ( Kim *et al.*, 2021: 630; 633)
- finding temporary measures for school exams, favouring a way to protect exams integrity that caused further inequalities of education attainment ( Arar *et al.*, 2024; Kippin & Cairney, 2022a; Kippin & Cairney, 2022b)
- placing high administrative burdens on means-tested support for ‘underrepresented minoritized students (URM)’ ( Morgan *et al.*, 2021: 1; 10–11).
- exacerbating the poor housing conditions and lack of access to health services among migrant workers ( Istiko *et al.*, 2022).

Mudaly and Chirikure (2023: 3) describe the potential for COVID-19 to provide an impetus for ‘collaborative partnerships among public and private entities, scientists, donors, government departments and other bodies’. However, the favoured collaborative deepened ‘social instability and economic inequality’ via ‘economic exploitation’. Global North neoliberal models of STEM have been complicit in private companies making massive profits while delivering vaccines, favouring market dynamics over social justice and equitable distribution. This dynamic is part of the ‘neoliberal disease’ described by Sparke and Williams ( 2022: 15; see also Kheir-Mataria *et al.*, 2023; and Ahen, 2021: 165 on ‘the exercise of power that benefit the major global health actors at the expense of stakeholders’).

These examples continued after initial lockdowns and attempts to return to normal life. For example, Matsumoto and Viczko (2023) describe the Canadian government’s use of a COVID-19 policy window to prioritise a return to high economic activity, such as to attract financially valuable international students from select countries (expanding the *Student Direct Stream* to students from South American coiuntries). The scheme was a financial quick fix and, “not aimed at providing equitable access to in-person learning in Canada to international students across the globe since it only provides a faster application process for ‘select’ students who are residents of particular countries” (2023: 1124). They cite Molla and Cuthbert (2023), who describe the Australian government using the COVID-19 crisis in a similar economy-focused way, to reflect their high reliance on international students and reinforce their ‘neoliberal’ and ‘human capital’ approach to higher education focusing on external income generation, economic productivity, and only relating equity concerns to urban/rural (regional) divides and some support for Indigenous students.

### 4. We should not underestimate the difficulties of generating usable lessons or policymakers using lessons

For COVID-19 equity researchers, an ideal policy process would exhibit a clear and direct link between: research drawing on insights from academics (and relevant social groups) to identify problems and potential solutions (sections 1 and 2 in Results), and the high uptake of evidence by a cadre of policymakers determined to devote proportionate attention and resources to the problem of COVID-19 inequity. This process would include policymakers devoting attention to demonstrable success from elsewhere, trying to solve the problem of health inequity (defined in the same way by both relevant governments), and adapting external experiences to their different contexts. However, a wealth of research on policy failure identifies learning resistance linked to narrow frames of reference, limited attention to contemporary evidence, and a tendency to internalise learning within an elite group at the expense of ‘politically weak minority’ voices that could inform more equitable approaches ( Dunlop *et al.*, 2020: 369–70; Dunlop, 2017). These dynamics contribute to the following barriers to policy learning for equity.

***Policymaker attention to health equity is overshadowed by health protection and security***

As with our previous reviews, we find researchers describing high urgency to address a profound and unfair inequalities problem, but low traction for equity ideas in policy processes. Their calls to see health equity through a social determinants lens were answered rarely. Indeed, governments were even slow to gauge the unequal impacts of COVID-19 and COVID-19 policy ( van Schalkwyk & McKee, 2021). Further, this framing of COVID-19 was overshadowed by a focus on health protection and security, often combined with a dominant economic framing of policy priorities ( Henson *et al.*, 2020).

Holst and van de Pas ( 2023: 1, citing Aldis, 2008; Wenham, 2019) describe the negative impact of such higher profile health security frames, which reinforce an ‘already dominant biomedical understanding of global health and the securitization of health in foreign policy’. The health security frame contributes to ‘technocratic reductionism’ at the expense of health promotion or attention to the social determinants of health and unequal spread of NCDs. As such, it ‘neglects root causes of global health crises … social, economic, political, commercial and environmental determination of health’ (2023: 1). A dominant focus on health protection (NPIs then vaccines) exaggerates the sense that we are all in it together, when really the ability to benefit from NPIs, and deal with the spread of disease and susceptibility to harm (linked to NCDs and underlying conditions) is profoundly unequal. It also exacerbates a tendency for country governments to protect their nation state from external threats rather than contribute to global policies for public health, thus contributing to a lack of serious attention to domestic and global equity (2023: 2).

This securitization of COVID-19 exacerbates longer term trends in health security. Aldis (2008) describes health security as an ambiguous and contested concept that can harm international public health cooperation (or become devalued when it means everything and therefore nothing – Wenham, 2019: 1097). Only for some does it represent an extension of global public health ideas that highlight the wider social determinants of health and the underappreciated importance of non-communicable diseases: ‘a primary health care approach, emphasizing community involvement, self-sufficiency and protection of vulnerable groups such as pregnant women and the poor’ (2008: 373). However, for others it is an extension of wider security frames focusing on:

1. counteracting external threats (including bio-terrorism), in this case from the spread of disease associated with epidemics in other countries
2. providing foreign assistance that combines health and military personnel, such as in ‘failed states’ or states threatened by terrorism, where the safety of health workers is threatened, and
3. joining up foreign policy with development funding and public health aims (2008: 371–72; Wenham, 2019: 1101–4).

It is common to find vaguely expressed aspirations to take a WG approach to such issues, emphasising the benefits of coherence and integration through consensus seeking ( Cairney, 2025). However, in this case, we see conflict in priorities in WG approaches. Examples include countries becoming suspicious of the motives of others, such as during population health surveillance, or engaging in UN-level contestation on ‘global health security’, such as when economically developing countries see key measures as a way for developed countries to protect their national interests without helping others (e.g. when sharing data on viruses to inform vaccine production) ( Aldis, 2008: 372). Or, relatively high funding for rapid emergency responses may come at the expense of longer-term preventive or primary care focused on mitigating inequalities ( Wenham, 2019: 1107).

***Policymakers struggle to appreciate evidence from the experiences of other governments***

Policymakers may only learn lessons about the issues they care about, have some experience of, and can understand in relation to their narrow perspective. Hence, they may ignore lessons about the social groups that are out of sight and mind or country experiences that seem alien to their way of life.

For example, An *et al.* (2021: 1167–68) identify a gulf between the policies that they find to be unambiguously effective when adopted – such as mask mandates – and the low propensity to use such evidence-informed responses, particularly when the measure would challenge government ideology or exacerbate the public cultural factors that breed resistance to such changes.

In many cases, this lack of lesson-learning relates to Western countries not learning from South East Asian countries:

‘East Asian countries recent and direct experience of previous outbursts of contagious pathogens … mediated their responses to COVID-19. With little direct or recent experience of a pandemic … the west struggled to act, raising the possibility that for policy lessons to ‘stick’ the learning process must be experiential’ ( Dunlop *et al.*, 2020: 369)

For example, An and Tang (2020: 790) identify a three-part story of the relative early success of ‘five advanced economies in East Asia: Taiwan, Hong Kong, South Korea, Singapore, and Japan’.

First, under the right conditions, there is some hope for countries to be better prepared after crisis. Taiwan, Hong Kong, South Korea, Singapore had developed infrastructures to deal with the COVID-19 epidemic following their experience with SARS (Severe acute respiratory syndrome) and MERS (Middle East respiratory syndrome), including: new emergency measures for containment, surveillance, long-term public health behaviour campaigns to foster norms on routine mask wearing, and – in Taiwan, Hong Kong, and Singapore - heavy punishments and policing for non-compliance with isolation measures (2020: 795; 797). Similarly, Moon (2020: 652–3) describes the profound – and eventually positive - effect of a South Korean government failure to deal with MERS, prompting major reforms ‘so as not to forget the failure and to turn the painful experience into useful guidance in handling future similar cases’. Reforms included to boost the status, autonomy, and capacity of the Korean Centers for Disease Control and Prevention (KCDC), and accept the need for high transparency during communication, which allowed the Korean government to pursue major state intervention, including mass testing and surveillance and isolation, while maintaining public trust and compliance (2020: 653–5).

Second, however, the scope for learning and change in these countries may not be transferable to Western liberal democracies exhibiting different norms of state intervention:

‘Policy instruments that infringe on individual freedom are more feasible and sustainable in East Asian culture that emphasizes collectivism. The culture governs social behavior with strong social norms and rules, is less tolerant of deviant behavior, and emphasizes the sacrifice of individual freedom for the collective good dur ing a crisis. In western culture, by contrast, where individualism prevails, stringent policy instruments may not be sustainable over a long time when public cooperation and voluntary compliance dwindle. Hence, policy instruments that work in East Asia may not work well in other countries’ ( An and Tang, 2020: 791)

Third, some experiences of relative success hide problems regarding a lack of equitable treatment. For example, Singapore demonstrated a whole population problem emerging from a lack of attention to major inequalities: ‘major outbreaks emerged among migrant workers … living in the government-run dormitories’, contributing to problems with general COVID-19 containment (2020: 797). In this case, the lesson regarded partial policy failure prompting attention to a moral case to address ‘the special needs of marginalized or vulnerable groups such as low-income foreign workers, who may require additional resources, such as free testing and additional government surveillance, to help remedy their disadvantaged circumstances’ (2020: 797).

Overall, we find different lessons - positive on general effectiveness but mixed regarding inequalities - in the context of limited overall comparability and a low propensity to learn (e.g. UK government actors expressly ruled out direct lessons from countries like South Korea, Cairney, 2021).

***The usability and political feasibility of lessons from research is often limited***

There is a major and well-documented issue regarding most research produced by academics and its limited use or usability by policymakers. Contemporary discussions highlight the unmet need to get away from a ‘linear’ model, where researchers simply send research to policymakers without considering their audience, towards ‘relational’ or ‘systems’ approaches in which academics and policymakers have a better mutual understanding of each other’s worlds and the constraints in which policymakers operate ( Best & Holmes, 2010; Oliver *et al.*, 2022). This unmet need is accentuated by COVID-19 research that offers vague lessons from initial experiences of success without considering what caused success or how it could be transferred to other governments (e.g. Powell, 2023; Powell & King-Hill, 2020; Cairney, 2025). Considerable evidence exists *on* policy, including some on its impact on inequalities (e.g. Jackson *et al.*, 2022; citing Hale *et al.*, 2021; see also Apostol *et al.*, 2022), but it is not clear how it would be used *for* policy.

Three included studies demonstrate this continuing gulf between research and policy by highlighting the kinds of evidence that policymakers find usable, and the considerable steps required to make this evidence work, technically and politically, in relevant networks ( Bandola-Gill, 2021; Bates *et al.*, 2023; Trapero-Bertran *et al.*, 2022).

Bandola-Gill (2021) compares how two international organisations try to secure the widespread use by governments of their measures of global poverty: (1) the World Bank’s US Dollar measure and (2) UNICEF’s multiple measures that are akin to social determinants (‘including eight dimensions of deprivation: education, health, nutrition, water, sanitation, shelter, information, and protection from violence’, 2021: 622). In each case, organisations are trying to present their knowledge as ‘legitimate’, maintain the high ‘status’ of their experts, and navigate ‘the rules or systems governing evidence use’ (2021: 619). In other words, to ensure the usability of their research, their measures had to enjoy technical and political feasibility: to be seen as driven by objective expertise but in a form that is acceptable to enough policymakers when deciding whether to use it. For example, the World Bank maintained two separate teams, with one on the technical side and the other maintaining political relationships to make sure that governments would not reject their approach (e.g. if measurement changes made governments and their policies look bad). UNICEF produced more complicated and challenging measures, but then worked with each government to help make those measures useful to their audience (2021: 628–30).

Bates *et al.*’s (2023) study - of research on the wider determinants of health – shows that the dynamics of complex policymaking systems accentuate this difficulty of navigating the rules of evidence use. Here, the problem is multiplied by the scale of the task of associated with ‘wicked’ public health issues that require engagement across multiple sectors and levels of government. Suppliers of research evidence face the need to engage with many audiences, spread across policy sectors, governments, and outside of government (see the reviews by Cairney *et al.*, 2021; Cairney, 2025). Many of these audiences have distinctive expectations for evidence usability, such as to favour quantitative over qualitative data (or some mix of both) when emphasising evidence credibility, or to relate evidence to different aims when using it instrumentally (e.g. private businesses valuing data to demonstrate the economic value of healthier living) (2023: 386). Further, organisational rules or standard procedures may limit their ability to incorporate certain types of evidence, such as when relatively abstract health aims are overshadowed by more concrete ‘legal and regulatory requirements to which private firms are subject’ (2023: 402).

Bates *et al.* (2023: 386) also identify a common theme across sectors: ‘actors revealed a strong preference for narratives with a strong emotional impact, supported by credible evidence’ (which chimes with True *et al.*’s, 2007: 161 summary of interest group framing strategies, which involve ‘a mixture of empirical information and emotive appeals’). This point should not be exaggerated, since we are not talking about a heroic narrator winning the day with a great personal story without evidence of wider relevance. Rather, stories resonate when they bring evidence to life, to help to explain real-world implications and make evidence more understandable or relatable, such as when measuring the impact of urban policies on health (e.g. in relation to transport and pollution) or the economic costs of poor health (e.g. lower productivity - 2023: 398–9).

The overall lesson is that most research on COVID-19 and equity may be routinely ignored if its authors or advocates are unwilling or unable to engage with multiple policymaker audiences well enough to understand how they will use it. Three factors explain this risk of speaking truth to power in an empty room. First, the routine obstacle is a tendency for academics to prioritise the research methods to produce the work and/or the challenging message to emerge, rather than the political policymaking context in which actors use information. Second, researchers struggle to navigate what Cairney and Toth (2023) describe as a tension between insider interest groups strategies and science advice principles: the former emphasises following the rules of the game to ensure inclusion in policy processes; the latter emphasises the need for scientists to remain credible among their own profession, with reference to independence, transparency, and critical distance. Indeed, this dilemma for researchers – to choose lower independence and higher influence or vice versa – prompted some frustration among the scientists who were critical of government policy from a distance (e.g. McKee *et al.*, 2022).

Third, these dynamics vary considerably by audience in terms of their expectations for evidence quality, their use of evidence for political or operational purposes, and the extent to which the evidence resonates in terms of their emotional and cognitive shortcuts. There are routine obstacles to policy learning in complex systems that comprise multiple actors, spread across multiple venues, and cooperating or competing to determine what evidence and lessons are relevant to what decisions ( Bates *et al.*, 2023: 403). In short, the demand for COVID-19 evidence or lessons is unclear and contested, and there is not one centralised means to resolve this contestation (2023: 393–4).

## Discussion

‘COVID-19 offers a unique opportunity to conduct truly global large N comparative studies of the processes and forces that underpinned pathological policy responses. Most obviously, this links to questions concerning the limits and possibilities of learning as we compare cases of failure with success’ ( Dunlop *et al.*, 2020: 369–70).
  ‘Lessons about successful transfer involve a clear idea of policy success, understanding how the policy instrument or mechanism links with success in the original context, and how ‘fungible’ it is to the new context. Put another way, the ‘COVID lessons industry’ may itself need to learn that lessons about policy transfer should be informed, complete and appropriate’ ( Powell, 2023: 88).

Potentially, there are many lessons to learn from pandemic policy experiences, and a huge research literature erupted on the topic of COVID-19 policy. However, most of that research identified unfulfilled aspirations for policy and showed little understanding of the practical and political realities of policymaking. From the perspective of researchers, policymakers are not sufficiently motivated to address the problem of inequity, even when the COVID-19 pandemic exposed profound inequalities. From the perspective of policymakers, researchers struggle to communicate lessons that are usable or solutions that are politically feasible. Therefore, there is a very realistic prospect of a huge expansion of academic research on COVID-19 and equity that translates into minimal impact on government. Unless we learn properly from COVID-19, its legacy will be false hope, repeated mistakes, and unequal outcomes.

These findings connect strongly to previous results from our wider series of *Open Research Europe* reviews of equity and policy research (three are included in this review – Cairney *et al.*, 2021; Cairney *et al.*, 2022b; Cairney & Kippin, 2022; see also Cairney *et al.*, 2023). Indeed, we find the same broad narrative in HiAP (Health in All Policies) research, which focuses on the limited adoption of global public health agendas that use a social determinants lens and focus on population-wide or ‘upstream’ policy measures:

‘HiAP research tends to engage in a circularity of enthusiasm and disappointment: (1) identifying the need for radical policy change, (2) promoting a new and ‘evidence based’ strategy to be adopted by each government, then (3) identifying implementation gaps, relating them to low political will, and expressing disillusionment with the politics of policymaking, before (4) restating the need for radical policy change’ ( Cairney & Kippin, 2022: 22).

In that context, we connect the current review to three broad insights from policy theories to identify more realistic expectations for policy and policymaking. First, minor policy change is the norm, and major policy change is rare and unpredictable. Further, ‘policy’ tends to describe a large mix of existing instruments to determine tax and spending, regulation, the allocation of public service resources, and provide information and guidance. As such, a major change in one strategy or instrument does not signal major change overall, and we should beware equating policy change on paper with its likely translation into practices and outcomes ( Cairney *et al.*, 2021: 23). These insights prompt uncertainty and unresolved debate on how transformative policy change could or would happen: would we expect something akin to a rapid pressure dam effect, or more gradual transformation in a series of changes ( Cairney *et al.*, 2023: 24–5)? Here, we find a contrast between the vaguely hopeful positions of COVID-19 equity scholars using policy theories to narrate their push for change versus policy theory scholars emphasising our lack of ability to fully conceptualise far less predict such dynamics.

Second, the gap between research evidence and policy is a routine feature of policymaking. Facing ‘bounded rationality’ ( Simon, 1976), policymakers can only pay attention to a small fraction of their responsibilities and the available information on those issues. This limitation prompts research on two very different issues: uncertainty and ambiguity. Researchers often focus on reducing uncertainty by providing more information, but policymakers seek ways to minimise their attention to information, such as by drawing on very limited and trusted sources. Further, the main driver for policy actions regards ambiguity: the ability to interpret the same problem in competing ways, producing competition to draw attention to one frame over others ( Cairney & Kippin, 2022: 23–24). Here, we connect these dynamics to COVID-19 equity research insights on policy learning, to recognise a tendency for policymakers to pay low attention to inequalities, reject the argument that they are necessarily unfair (many portray marginalised groups as undeserving of further support), pay more attention to economic and security frames, and limit the extent to which they learn from governments exhibiting different perspectives and ways of working. The provision of more research does not mitigate this problem.

Third, these dynamics take place in complex policymaking environments that are outside of policymaker control. There is no single centre of authority. Rather, policy theories try to conceptualise the emergency of policy outcomes from environments characterised by:

- 'Many policymakers and influencers spread across multiple levels of government ( *actors*).
- Multiple venues for authoritative choice, each with their own informal and formal rules ( *institutions*).
- Relationships between the actors responsible for making policy and those who influence and deliver it ( *networks*).
- Dominant beliefs and assumptions about the policy problem ( *ideas*).
- The socio-economic factors and events that influence policymakers and are out of their control ( *policy context or conditions*)’ ( Cairney & Kippin, 2022: 25).

This insight is crucial to the use of policy theories to anticipate - as well as explain - change. Much like in HiAP research, COVID-19 equity scholars use policy theories to narrate optimistic stories of agency and policy change, in which policy entrepreneurs can help to win the day, new coalitions can form to challenge previous dominant coalitions, or the old ways of doing things are punctuated and replaced with new ways of working ( Cairney *et al.*, 2021). In this COVID-19 review, the equivalent optimism comes from the idea of exploiting new windows of opportunity for the adoption of policy changes and similar conceptions of policy punctuations. Yet, in policy theory narratives, there is far less emphasis on the agency of key actors or their ability to harness periods of policy change. Rather, key actors are adapting relatively well to environments beyond their control rather than reshaping those environments ( Kingdon, 1984 described skilful surfers, not water gods). Further, as the research included in this review suggests, even the most skilful and persuasive equity policy entrepreneurs would struggle to boost the political feasibility of measures to reduce inequalities if they challenge dominant ways of thinking and working during a period of crisis. Here, the crucial distinction is between a crisis associated with events versus a required (but generally absent) crisis of confidence in existing approaches (e.g. see Hall, 1993).

Cairney *et al.* (2022b) translate these insights into three requirements to boost policy change to address inequalities:

1. *Clarity*: improve the meaning of COVID-19 equity. Our review highlights two types of ambiguity undermining progress. The first regards contestation within public health research about the meaning of health equity. High conceptual ambiguity among key actors (like the WHO) provides insufficient direction and a limited means to evaluate progress. The second regards ongoing contestation to decide which inequalities are unfair, and what issues and social groups to prioritise. In each case, improving clarity, involves ongoing political contestation rather than consulting a dictionary.
2. *Congruity*: improve the connection between equity aims and routine government business (which includes a tendency towards short-termism). Our review highlights some hopes that policymaker attention would lurch towards radically new policy solutions, but without a clear sense of how they would relate to current commitments. Or, we find a win-win case for change, to emphasise the wider economic benefits to state intervention to improve public health. In each case, there is unresolved uncertainty about how to relate radical policy change to the status quo.
3. *Capacity*: improve the direction of resources to the policy problem. It is common in public health, and wider equity research, to bemoan the lack of ‘political will’ among policymakers responsible for change. We also emphasise the need for systemic capacity in government, such as to ensure that policymaking organisations devote significant effort to equity aims ( Cairney *et al.*, 2022b: 7). Our review highlights a general absence of attention to how policymaking systems would change to accommodate policy reforms. In other words, what exactly is there a window of opportunity for?

## Conclusion

COVID-19 prompted many scholars to reimagine a more equitable world. In this scenario, the crisis induced by COVID-19 would provide policymakers with a window of opportunity for radical changes to address longstanding inequalities. Indeed, governments had shown a willingness and ability to intervene rapidly and substantively to address a crisis in public health, so why not harness that energy to address the health inequalities that became so stark during the pandemic? We set out this narrative in four main parts.

First, although governments were slow and often reluctant to gather data systematically, there is clear and substantive evidence of unfair COVID-19 inequalities. The impact of the pandemic, and most government responses, exacerbated unfair and preventable inequalities in relation to factors such as poverty, race and ethnicity, gender, and disability. These factors are familiar to researchers of the unequal spread of NCDs which contributed to the pre-existing conditions that increased susceptibility to COVID-19 harm. Both the unequal spread of NCDs and COVID-19 impact highlight the profound contribution of social and economic factors to ill-health. Further, the unequal COVID-19 experience contradicts an often-espoused idea that we were all in it together. Rather, the spread and impact of COVID-19 depended heavily on factors such as age, income, race, gender, and disability and the willingness and ability of governments to mitigate their unequal effects (such as to foster equal access to essential public services, vaccinations, and support for financial stress).

Second, there was some hope that COVID-19 would be the crisis to open a new window of opportunity for policy change. If a pandemic and pandemic response can shine more light on longstanding inequalities and the social determinants of health, there is high potential to generate attention to the problem of inequity then answer the call for new policy solutions. Hence, we find a wealth of research that identifies many large and pressing policy problems and a collection of potential policy solutions to be considered during the COVID-19 policy window. They include a broad push for greater state intervention to boost the social and economic resilience of populations and public services rather than see resilience as an individual trait, and specific measures to be better prepared for crisis, such as to boost capacity to shift education, health, and social services online.

Third, however, this research evidence signals a major gap between such aspirations and reality. In most cases, a policy window opened but closed before ambitious and equitable solutions became technically and politically feasible (or policymakers made vague commitments with little prospect of delivery). These obstacles were particularly stark when policymakers adopted individualist over social determinants lenses, and when right-wing parties rejected solutions that required state intervention. In some cases, the window of opportunity opened to adopt policies that promoted population health security while exacerbating inequalities.

Finally, we should not underestimate the barriers to generating and acting on usable lessons from this research. Before COVID-19, a focus on health improvement and social determinants was usually overshadowed by the dominant lens of economic security. During the pandemic, the lens of health security also took most attention from health equity. Further, although there are stories of relatively successful government responses, it is difficult to know exactly how policy worked well elsewhere and if the same approach would work here. For example, Western liberal democratic governments appeared unwilling or unable to extract usable lessons from successful governments in Southeast Asia. At the same time, there is a major disconnect between the *audience for lessons*, such as policymakers paying fleeting attention to inequalities, and the *source*, such as the academics struggling to communicate research in a form that is usable for policymakers. For example, some studies explain the considerable lengths to which international organisations go to have their research used by policymakers, and these practices contrast with those of most research organisations focused more on maintaining their professional credibility and critical distance from government, thus contributing to the lack of politically feasibility of most options to emerge from academic COVID-19 equity research.

Overall, the included and snowballed research demonstrates that health inequalities and their social and economic determinants had a profound impact on COVID-19 (and vice versa), but they remained a low priority for most governments. Attention to inequalities related to disability, race and ethnicity, and gender grew from a low base, then struggled to compete with a more general ‘we are all in it together’ message. Resources for health improvement were redirected to health protection. Concerns for the economy overshadowed concerns for economic inequalities. The new normal quickly came to resemble the old normal. Most windows of opportunity opened and closed without substantive positive change. In that context, while COVID-19 prompted scholars to reimagine a more equitable world, they expect that most governments will contribute to similar unfair inequalities in their responses to the next crisis.

## Ethics and consent

Ethical approval and consent were not required.

## Data Availability

No data are associated with this article. Open Science Framework: COVID19 equity policy ( Cairney & Intropido, 2025) 10.17605/OSF.IO/UE36C ( Cairney, 2025). This project contains the following extended data: - Structured bibliography https://osf.io/nbygf - Study Protocol https://osf.io/y4zkh Data are available under the terms of the Creative Commons Attribution 4.0 International license (CC-BY 4.0) Open Science Framework: COVID19 equity policy https://osf.io/ue36c/ The project contains the following additional information: PRISMA checklist for the Open Research Europe version (page numbers refer to submitted Word version) https://osf.io/xck5w PRISMA flow diagram: https://osf.io/fqa4k Data are available under the terms of the Creative Commons Attribution 4.0 International license (CC-BY 4.0)
