# Supplementary material for: The pursuit of equity in COVID-19 policy and policymaking: A qualitative systematic review
Source: Open Res Eur. 2026 Jun 23;5:167. Originally published 2025 Jun 18. [Version 2] doi: 10.12688/openreseurope.20563.2 (PMC13324367; doi:10.12688/openreseurope.20563.2)
Supplement: Peer review history

# Reviewer report: Reviewer response for version 2

**DOI:** 10.21956/openreseurope.26328.r76332

**Published:** 2026-07-01

**Authors:** Isabella M Radhuber

**Affiliations:** 1 University of Vienna, Wien, Austria

The authors appreciated the comments and addressed them throughout the paper, improving clarity, foregrounding their argument in more direct ways and clarifying open questions such as explaining biases regarding authors from the Global North and the Global South. Based on this assessment, I support the indexing of the article and appreciate the opportunity to engage with and learn from their paper.

Are sufficient details of the methods and analysis provided to allow replication by others?

Yes

Are the rationale for, and objectives of, the Systematic Review clearly stated?

Yes

Is the statistical analysis and its interpretation appropriate?

Yes

Are the conclusions drawn adequately supported by the results presented in the review?

Yes

Reviewer Expertise:

Isabella M. Radhuber is a senior researcher specialising in environmental, climate and health politics from a transdisciplinary perspective. Trained as a political scientist, she explores the political dimensions of global crises, decolonisation, and multidimensional inequalities, with a particular focus on climate and health.

I confirm that I have read this submission and believe that I have an appropriate level of expertise to confirm that it is of an acceptable scientific standard.

# Reviewer report: Reviewer response for version 2

**DOI:** 10.21956/openreseurope.26328.r76331

**Published:** 2026-06-26

**Authors:** Geoff Bates

**Affiliations:** 1 University of Bath, Bath, England, UK

No further comments.

Are sufficient details of the methods and analysis provided to allow replication by others?

Yes

Are the rationale for, and objectives of, the Systematic Review clearly stated?

Yes

Is the statistical analysis and its interpretation appropriate?

Yes

Are the conclusions drawn adequately supported by the results presented in the review?

Yes

Reviewer Expertise:

Public health policy, use of evidence and research in and for policy, qualitative and evidence review methods, systems approaches.

I confirm that I have read this submission and believe that I have an appropriate level of expertise to confirm that it is of an acceptable scientific standard.

# Reviewer report: Reviewer response for version 1

**DOI:** 10.21956/openreseurope.22248.r55842

**Published:** 2025-08-22

**Authors:** Isabella M Radhuber

**Affiliations:** 1 University of Vienna, Wien, Austria

In this article, the authors examine how COVID-19 equity research assessed the policy problem, solutions and their feasibility in a complex environment. They conducted a qualitative systematic review in 2024 and identified 55 peer-reviewed articles and 30 additional studies, all of which addressed COVID-19, policymaking and equity in a meaningful way. The literature documents how the pandemic and government responses have exacerbated existing inequalities and describes a range of equity-oriented policies that it is hoped will be adopted.[1] However, the research also reveals a wide gap between the desire for change and the political reality: obstacles in the political processes limited the learning process and the adoption of lessons on equality. The authors conclude that most governments have learned few lessons from health inequalities, and that without sustained attention, similar inequitable outcomes are likely to be repeated in future crises.

 1.) Comments regarding the readability, narrative, and presentation

 As it stands, the review is a highly valuable contribution, focusing on policy processes that explicitly consider inequalities in the context of the COVID-19 crisis – a topic that is both highly relevant and under-researched in the face of today’s intersecting crises. However, I believe that the narrative can be a little more integrated. The authors describe two conflicting perspectives, but in their/your own words, can you express in one sentence what this tension is all about? There are so many interesting, crucial and important details in your analysis, and such a sentence could make it easier for the reader to grasp the relevance of your research/review.

 Although the review is clear enough in its current version, I believe that both the introduction and the empirical chapter could begin with a stronger, simplified and engaging narrative. (For example, the introduction now begins as follows: „We present a qualitative systematic review of COVID-19 equity policy research. We focus on how researchers and policymakers define unfair inequalities and seek to improve equity, and what governments can learn about reducing inequalities when planning for the next crisis.”) This might make it easier for the reader to engage with the topic early on.  It gets more exciting towards the end, and the narrative could be strengthened at the beginning of the sections.

 To strengthen the narrative, this detail the authors/ you mention in the review might be a good starting point: “In this scenario, the crisis induced by COVID-19 would provide policymakers with a window of opportunity for radical changes to address longstanding inequalities.”; and “Further, very few studies of policy processes and the COVID-19 crisis focused specifically on inequalities or the inequitable experiences faced by marginalised social groups ( Bromfield & Carey, 2025).” Perhaps this gap could be made clearer from the outset? This would be a strong start.

 Some issues that are implicitly referred to in the text could be taken up more explicitly; these include:

- The need for a contextualised policy approach (see e.g. third paragraph: : “This contestation extends to policy evaluation, including the measures that we use to determine success ( McConnell, 2010). The ability to transfer success from one territory to another is fraught with difficulties, such as when seeking to identify: the evidence for (and cause of) one government’s success, if an importing/exporting government defines and addresses the same policy problem in the same way, and if their political and policymaking systems are sufficiently comparable ( Cairney *et al.*, 2022a). Therefore, the kinds of policy learning envisaged in the first perspective are not routine features of policymaking“),
- the tension between a bottom-up and a top-down policymaking approach,
- or the role of coloniality.

2.) Comments regarding the methodological presentation

 Overall, the methods are very thoroughly applied and described in great detail. I have one question and one suggestion:

- Regarding the methodological design, it is not clear to me why the open access filter was used. Could the authors/ you please explain whether this means that you only included open access papers, and if so, why this decision was made? This could be important considering that the decision to go OA often depends on unevenly distributed funding opportunities.
- The authors/ you mention that “an insistence on citing mainstream policy theories from the Global North would exclude useful articles produced in the Global South”, reflecting this in part. However (and since this review focuses on inequalities) this point could be expanded just a bit further. This has to do with existing inequalities and how and by whom inequalities are examined (invisibly or visibly). Perhaps a sentence should be added here to explain why this might be the case. What are the obstacles? What efforts are being made to overcome them? How could reviews be conducted differently to address these inequalities?

3.) Referring to discussions about political theory and “the political”

 As the authors/ you discuss how policymakers and researchers can learn from each other, I think a number of (inter)disciplinary debates can be referenced that are relevant in this regard, such as political science discussions on “the political” and the tole of knowledge, values, interests, power, lobbies, etc. While policy theory scholars are mentioned, there is no further discussion of this work (or at least references to ongoing debates). This discussion could also be linked to a call for transdisciplinary research, for example.

 (There is more political science literature on this, for example: “For COVID-19 equity researchers, an ideal policy process would exhibit a clear and direct link between: research drawing on insights from academics (and relevant social groups) to identify problems and potential solutions (sections 1 and 2 in Results), and the high uptake of evidence by a cadre of policymakers determined to devote proportionate attention and resources to the problem of COVID-19 inequity.”)

 4.) Briefly introduce the previous reviews

 The previous reviews written by the authors are mentioned a few times, arousing curiosity as to what they are about and where they can be accessed; I suggest adding this information. I also suggest adding some explanation of what the “resulting consolidated approach” is.

 5.) Refer to open questions

 Although I understand that this may be beyond the scope of this review, I would personally be interested in reading about the blind spots, open questions, and research gaps that the authors identify across disciplines.

 6.) Some formal details.

 In addition, I believe I have found some minimal formal details that need to be corrected:

- Make sure that COVID-19 is spelled the same throughout the text (once it is COVID, once it is COVD-19).
- I think, some words are missing here: “For example, Kheir-Mataria et al.’s (2023: 4) Delphi panel describe facilitators of equity as ‘better representation of countries from the global south’, ‘a mechanism to monitor the influence of private actors and non-governmental financing organizations in policymaking’ (to address vaccine profits and inequitable access), ‘change the role of the World Health Organization’ and foster more powerful and effective global health governance and accountability.”
- There’s a word missing here: “However, the favoured collaborative deepened ‘social instability and economic inequality’ via ‘economic exploitation’.”
- Delete the excessive space here: “The culture governs social behavior with strong social norms and rules, is less tolerant of deviant behavior, and emphasizes the sacrifice of individual freedom for the collective good dur ing a crisis.”
- I think this sentence needs correction: “Hopes for reform come also come with uncertainty and debate about the best governance model to support progress. For example, Koon et al. (2021: 1) conclude that regulations/mandates are essential in the US, since only providing guidance or appealing to voluntarism would not be effective with people who see COVID-19 as a conspiracy or behavioural change as a threat to their livelihood.”
- I think this sentence needs correction: “Here, the problem is multiplied by the scale of the task of associated with ‘wicked’ public health issues that require engagement across multiple sectors and levels of government”
- I think this sentence needs correction, and/or could be simplified: “For example, some studies explain the considerable lengths to which international organisations go to have their research used by policymakers, and these practices contrast with those of most research organisations focused more on maintaining their professional credibility and critical distance from government, thus contributing to the lack of politically feasibility of most options to emerge from academic COVID-19 equity research.”

[1]  The empirical results are presented in four thematic subsections, each containing a selection of topic groups: “This research (1) identifies the unequal impact of the COVID-19 pandemic and policy, then (2) expresses some hopes for policy solutions to support more equitable processes and outcomes. However, it (3) highlights a major gap between spiration and reality (or the proposal versus acceptance of such solutions), and (4) identifies barriers to the production and use of lessons for future crises.”

Are sufficient details of the methods and analysis provided to allow replication by others?

Yes

Are the rationale for, and objectives of, the Systematic Review clearly stated?

Yes

Is the statistical analysis and its interpretation appropriate?

Yes

Are the conclusions drawn adequately supported by the results presented in the review?

Yes

Reviewer Expertise:

Isabella M. Radhuber is a senior researcher specialising in environmental, climate and health politics from a transdisciplinary perspective. Trained as a political scientist, she explores the political dimensions of global crises, decolonisation, and multidimensional inequalities, with a particular focus on climate and health.

I confirm that I have read this submission and believe that I have an appropriate level of expertise to confirm that it is of an acceptable scientific standard, however I have significant reservations, as outlined above.

# Response

**Published:** 2026-06-11

**Authors:** Paul Cairney

**Affiliations:** University of Stirling, UK

**Reply to Isabella M Radhuber** Thank you for your positive and constructive review. We have made the following revisions:

1. To add a new paragraph to each section (Introduction and Results) to strengthen the narrative (‘both the introduction and the empirical chapter could begin with a stronger, simplified and engaging narrative’).
2. To make key points more explicit and better explained: the importance of contextualisation (to which we refer in the Discussion); the tension between top-down and bottom-up perspectives on policymaking (which we make more explicit in the relevant section), and the role of coloniality (we explain the reference to ‘colonial logic’).
3. To make explicit the risk of bias to Global North authors by using Open Access as a filter in two databases (Limitations).
4. To clarify our inclusion criteria in relation to mainstream policy theories (Limitations).
5. To provide a brief initial explanation of the previous reviews and make more explicit our focus on ‘the political’ (in relation to knowledge, values, interests, power, etc.) (Introduction).
6. On the issue of identifying a gap in the literature, we add this section in the Discussion: ‘Here, we do not identify a gap in the literature, but rather the use of a policy theory literature in ways not anticipated by their original authors’.
7. To address the list of corrections identified at the end of the review. Thank you – we have made these corrections throughout the manuscript.

# Reviewer report: Reviewer response for version 1

**DOI:** 10.21956/openreseurope.22248.r56119

**Published:** 2025-08-18

**Authors:** Geoff Bates

**Affiliations:** 1 University of Bath, Bath, England, UK

This is a well conducted and reported study, with transparency and clarity on methods and full detail provided throughout. It demonstrates that Covid-19 and related policy responses contributed to widening inequalities, and that hopes that the pandemic would lead to a 'window of opportunity' for change were largely unmet in the face of substantial obstacles facing policymakers. It concludes that health inequalities were generally a low priority for policymakers and this is unlikely to change when governments are faced with future crises.

 The review nicely demonstrates the identified gap between hope and belief that experiences during the pandemic will lead to change and the political and governance realities of this happening.

 As demonstrated in my answers to the 4 questions, my report is a positive one with only a couple of minor suggestions to strengthen the work, for the authors to consider. I provide below a comment relating to each of the 4 questions in the report.

 Rationale and objective: clearly stated. The case for the review is well justified and its purpose and objectives are clear. One area of potential ambiguity is the use of terms equity, inequities and inequalities, which are key concepts for this review. The authors (rightly) highlight some issues around use of these terms and related ambiguity in the literature in the review results and discussion sections - would it therefore be appropriate to provide some clarity early on as to how they are using these terms themselves, since they are key to the review?

 Methods: Full and transparent reporting throughout, with consistency with methods in previous reviews in this series. I would almost argue there is too much detail on the search approach in the efforts for full transparency so that the reader could lost in the information, but it provides useful learning and reflection. If the authors agree, they might comment on the decision to limit some searching using the 'open access' filter given they recognise the bias of the research towards the global north. While the decision made seems sensible (and helped by the substantial snowballing approach to finding studies) I think it is worth acknowledging this where they discuss similar issues in the section 'limitations relevant to data gathering and analysis'

 Results: The approach to analysis is clear and transparent, and the section is very well presented and structured, and, importantly, interesting. The authors make many insightful points based on their analysis that each provide a useful addition to the literature. It is an ambitious synthesis of over 50 studies and consequently the section is lengthy - I would argue perhaps overly so, as there is so much for the reader to take in so that important points could be lost - particularly I felt in finding headings 2 and 4. With that said, it all feels well justified within the scope of this review and there is a strong narrative throughout. At over 8,500 words my view is that it is worth the authors reviewing the section with a critical eye for reducing text where they think possible to do so.

 Discussion and conclusions: 2 excellent sections that flow nicely from, and link clearly with, the analysis. The alignment with narratives in research on health in all policies works very well. The move to identifying realistic policymaking expectations is nicely done and provides important insights.

 Overall, I congratulate the authors on this substantial and well conducted and reported review.

Are sufficient details of the methods and analysis provided to allow replication by others?

Yes

Are the rationale for, and objectives of, the Systematic Review clearly stated?

Yes

Is the statistical analysis and its interpretation appropriate?

Yes

Are the conclusions drawn adequately supported by the results presented in the review?

Yes

Reviewer Expertise:

Public health policy, use of evidence and research in and for policy, qualitative and evidence review methods, systems approaches.

I confirm that I have read this submission and believe that I have an appropriate level of expertise to confirm that it is of an acceptable scientific standard.

# Response

**Published:** 2026-06-11

**Authors:** Paul Cairney

**Affiliations:** University of Stirling, UK

**Reply to Geoff Bates** Thank you for your positive and constructive review. We have made the following revisions:

1. To make explicit the risk of bias to Global North authors by using Open Access as a filter in two databases (Limitations).
2. To address the potential for key points to be lost in a lengthy Results section. We chose to retain most of the text, but added clearer signposts to the key points at the beginning of key sections, and boosted the section headings which correspond to the key points.  
